# Intersections of Sex Work, Mental Ill-Health, IPV and Other Violence Experienced by Female Sex Workers: Findings from a Cross-Sectional Community-Centric National Study in South Africa

**DOI:** 10.3390/ijerph182211971

**Published:** 2021-11-15

**Authors:** Rachel Jewkes, Minja Milovanovic, Kennedy Otwombe, Esnat Chirwa, Khuthadzo Hlongwane, Naomi Hill, Venice Mbowane, Mokgadi Matuludi, Kathryn Hopkins, Glenda Gray, Jenny Coetzee

**Affiliations:** 1 Gender & Health Research Unit, South African Medical Research Council, Pretoria 0001, South Africa; Esnat.Chirwa@mrc.ac.za (E.C.); coetzeej@phru.co.za (J.C.); 2 Office of the President, South African Medical Research Council, Tygerberg 7505, South Africa; Glenda.Gray@mrc.ac.za; 3 Faculty of Health Sciences, School of Public Health, University of the Witwatersrand, Parktown, Johannesburg 2193, South Africa; otwombek@phru.co.za; 4 Perinatal HIV Research Unit, Faculty of Health Sciences, University of the Witwatersrand, Johannesburg 2193, South Africa; milovanovicm@phru.co.za (M.M.); Hlongwanek@phru.co.za (K.H.); mbowanev@phru.co.za (V.M.); matuludimf@gmail.com (M.M.); 5 African Potential Foundation, Menlo Park, Pretoria 0102, South Africa; 6 Wits RHI, Faculty of Health Sciences, University of the Witwatersrand, Johannesburg 2193, South Africa; NHill@wrhi.ac.za; 7 Sabin Vaccine Institute, Washington, DC 20037, USA; kate.hopkins@sabin.org

**Keywords:** depression, PTSD, sex work, gender-based violence, stigma, rape, mental health

## Abstract

Female sex workers (FSWs) are at increased risk of mental health problems, including mood disorders and substance abuse, and we need to understand the origins of these to treat and prevent them, and particularly understand how the context in which they sell sex impacts their mental health. We conducted a multi-stage, community-centric, cross-sectional survey of 3005 FSWs linked to SW programmes in twelve sites across all nine provinces of South Africa. We interviewed adult women who had sold sex in the preceding six months, who were recruited via SW networks. We found that FSWs have very poor mental health as 52.7% had depression and 53.6% has post-traumatic stress disorder (PTSD). The structural equation model showed direct pathways from childhood trauma and having HIV+ status to mental ill-health. Indirect pathways were mediated by food insecurity, controlling partners, non-partner rape, harmful alcohol use, substance use to cope with SW, indicators of the circumstances of SW, i.e., selling location (on streets, in taverns and brothels), frequency of selling and experiencing SW stigma. All paths from childhood trauma had final common pathways from exposure to gender-based violence (non-partner rape or intimate partner violence) to mental ill-health, except for one that was mediated by food insecurity. Thus, FSWs’ poor mental health risk was often mediated by their work location and vulnerability to violence, substance abuse and stigma. The potential contribution of legal reform to mitigate the risks of violence and mental ill-health are inescapable. Treatment of mental ill-health and substance abuse should be an essential element of FSW programmes.

## 1. Introduction

Globally, mental ill-health is a serious public health concern, frequently occurring concurrently with other physical illness [1]. In most countries, female sex workers (FSWs) work in circumstances where selling sex is criminalised and discrimination is ongoing, and violence is an ever-present risk [2,3,4]. This severely undermines their mental health. Evidence from a study of FSWs working in Soweto, South Africa (n = 508), highlighted that 68.7% suffered from depression and 39.6% had PTSD [5]. A prior study in Kwazulu Natal Province (n = 115) also found high levels of depression and anxiety (78.4%) amongst FSWs accessing support services [6]. By comparison, in the South African general population, depression and high levels of PTSD symptomatology were found to be prevalent amongst 23% and 11.6%, respectively, of adult women, as revealed by a household survey in Gauteng Province [7]. A similar picture of poor mental health amongst FSWs has been described in other countries. The prevalence of depression in Nepal, India and Israel has been estimated at 82.4%, 39%, and 54%, respectively [8,9,10,11]. PTSD is less well documented, with Israeli and Australian research finding a prevalence in FSW populations of 31% and 17%, respectively [10,11].

**Figure 1:**
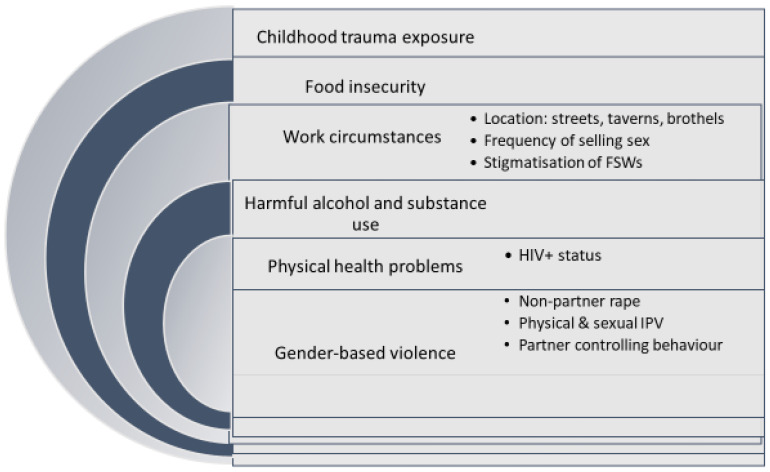
Conceptual model of factors associated with mental ill-health amongst FSWs.

The global burden of ill-health due to sex work, however, has not been described, despite calls for this to be a focus of research, not least because of the methodological challenges of conducting population-based surveys of FSWs [12]. The FSW population is hard to access through conventional survey approaches, and research has tended to draw on convenience or clinical samples or respondent driven sampling surveys in limited geographical areas. Drawing on South Africa’s first national study of FSWs linked to sex work programmes, we describe in this paper the prevalence of depression and PTSD among FSWs and, for the first time, explore the interconnections between a range of drivers of mental ill-health, including aspects of work circumstances. We analytically test a hypothesised conceptual model reflecting the complex interrelationship between drivers of mental ill-health amongst FSWs in South Africa (Figure 1).

In developing the model, our hypothesised life experiences associated with poor mental health is rooted in the published literature. Within the overall framework of criminalised sex work and the patriarchal social norms of South Africa, we hypothesise that FSWs’ mental health is influenced by factors that broadly stem from childhood experiences, poverty and food insecurity, circumstances of selling sex, the use of alcohol and other substances to enable selling sex, experience of physical health problems and gender-based violence, including partner controlling behaviour. The links between SW and mental ill-health often start from the point of entry into sex work. Women who are exposed to trauma in childhood, particularly child sexual abuse and emotional neglect, are much more likely to enter sex work than those who are not exposed, and sex is often sold before the age of 18 [13]. Women who have experienced child sexual abuse often experience dissociative disorders which assist them in being able to cope with being in sex work [14]. Another common coping strategy for many FSWs is alcohol drinking and other substance use [15,16], and both of these elevate the risk of being subjected to sexual violence, with the entailed trauma [17]. Sex workers are very highly susceptible to rape and intimate partner violence (IPV), and there is a greater risk for street-based as compared to indoor or venue-based SWs [18,19,20,21]. Women exposed to rape and IPV have a much higher likelihood of developing depression and, especially after rape, PTSD, and so it is plausible that mental health problems are associated more with work in particular settings [22]. Likewise, venue type is a driver of HIV risk, with street-based SWs being at greater risk of infection [23,24]. It is well established that childhood trauma, food insecurity, controlling partners and exposure to gender-based violence (GBV) are drivers of mental ill-health in the general population, and so are likely drivers among FSWs [7,25,26,27,28]. A 2018 study of FSWs in Soweto found that the age of first selling sex, internalised stigma and repeat violence exposure all increased the risk of either depression or PTSD, or comorbid disorders [5]. Exposure to violence in this population has been well documented.

In order to deepen our understanding of the prevalence of mental ill-health among FSWs and investigate the hypothesis that the groups of factors outlined in the figure are drivers thereof, and to investigate their interrelation, we present an analysis of the South African national survey using structural equation modelling (SEM). To our knowledge, this is a first attempt at developing and testing a model that encompasses such a broad range of potential drivers of the mental ill-health of FSWs.

## 2. Materials and Methods

### 2.1. Study Design and Setting

A multi-stage sampling procedure was used to derive a self-weighting sample, stratified by province. The recruitment phase drew on elements of RDS methods for hard-to-reach populations, which we had previously used in research with FSWs [29]. We stratified the sample by province and, for most provinces, one district was selected randomly. In the Northern Cape, we worked with a small outreach team based in one district. In KwaZulu-Natal and Gauteng Provinces, with larger numbers of SWs, we spread recruitment across two and three districts, respectively. The sample of FSWs to be interviewed per province was determined based on a 2013 SW population size estimation [30]. Seeds were invited to participate in the study, enrolled, and after data collection given three coupons to pass onto other FSWs in the district. We capped each referral chain at a maximum of 30 FSWs. The average recruitment chain was 9–10 women per venue. A full account of the methods and sample size calculation is available elsewhere [31].

For inclusion in the study, participants needed to be assigned female at birth and currently identify as female, be 18 years or older, work within the site’s district and to have voluntarily sold or transacted in sex for financial gain (not necessarily paid in cash) in the past six months. For ethical reasons, individuals who were <18 years, or who self-reported being a victim of human trafficking, were excluded from the study, but they were otherwise assisted.

### 2.2. Study Measures

Interview guides and questionnaires were developed in collaboration with SWs and SW peer educators and tested in cognitive interviews. The questionnaire was professionally translated into ten local languages. Each partner organisation’s sex work programme was asked to brief the FSW community (brothel owners and FSWs alike) in their district regarding the study to help ensure access and buy-in from key stakeholders. FSW peer educators were trained as interviewers. Data were collected from January to July 2019. Screening and informed consent was undertaken by a peer educator. Thereafter, participants completed a 40-min interviewer-administered survey. Participants were reimbursed for their time, to the value of approximately USD15.

We collected demographic information and the measures shown in Table 1. The questionnaire captured demographic details, including age, level of education (categorised into incomplete schooling versus completed secondary and any post school qualifications), and three items measuring food security (In the last four weeks, how often was there no food of any kind in the house due to lack of money, how often did you sleep hungry due to lack of food, and how often did you go a whole day and night without eating?). These were summed and then dichotomised to create some versus none. To determine whether women sold sex outdoors (‘on the street’), we asked a question that had 26 response options, any number of which could be chosen. We coded all women choosing any outdoor option (under a bridge, veld/field/bush, road/street, beach) as ‘selling on the street’. Those indicating that they sold sex in informal drinking establishments (shebeens/taverns) or in brothels were appropriately coded.

Women were asked the number of days they had sold sex in the past month. HIV status was determined by the results of two, concurrent rapid HIV tests (Abon™, First Response^®^ and Toyo^®^ tests) performed in the field or by laboratory HIV ELISA testing (if rapid results were indeterminate). Women were asked if they knew their HIV status and a variable was coded as indicating that they were HIV+ and knew this or else were HIV+ and did not know their status/were HIV negative.

### 2.3. Data Analysis

Data analysis was conducted in Stata 16.0, and all analyses took into account the design of the sample. Taylor linearization was used for calculating 95% confidence intervals. Univariate and bivariate descriptive statistics were calculated for categorical and continuous variables.

Structural equation modelling (SEM) was conducted to assess the interrelationship between variables in our conceptual model and mental health. We constructed a latent variable for the model outcome from the depression score and PTSD score. The correlation between each hypothesized variable and the mental health variable was then tested by building variable pairs. All associations were tested by running a full-information maximum likelihood method to deal with missing values. This method was chosen over multiple imputations as it has been shown to yield superior results in structural equation modelling [37]. To assess model fit of the observed data, we used the comparative fit index (CFI) (>0.95); Tucker-Lewis Index (TLI) (>0.95) as indicative of good fit [38]; and root mean square error of approximation (RMSEA) (of 0.05 or less) [39]. We tested the latent variables for convergent and divergent validity (acceptable validity (average variance extracted (AVE) > 0.5)). The main SEM was run in Stata 16.0, but a confirmatory analysis was performed using MPlus (MLR (Maximum Likelihood with Robust standard errors) and WLSMV (Weighted Least Square Mean and Variance adjusted) estimators).

We fitted a path model using full information maximum likelihood (FIML) estimation to model all available data. The final model was built based on theory and statistically meaningful modifications using backwards elimination to exclude putative variables that did not mediate any path (with significance set at the *p* < 0.05 level) from the exogenous variables to mental health in order to ensure model parsimony. We did not add covariances to improve model fit because the model fit was good (RMSEA, 0.044; CFI, 0.968; TLI, 0.956) and discriminant validity and convergent validity of the latent variables were good (AVEs: mental health 0.626; IPV, 0.696; squared correlation between mental health and IPV, 0.145). We present the direct, indirect and total standardized effects for each path.

### 2.4. Ethics Approval

This study received approval from the University of the Witwatersrand Human Research Ethics Committee (HREC) (Medical) (Ref number: 180809).

## 3. Results

### 3.1. Participant Demographic Characteristics

Table 2 summarises the participants’ demographic characteristics, and bivariate analysis showing the risk factors associated with mental ill-health amongst FSWs in South Africa. The mean age was 33.3 years, and 52.7% of women had depression (95%CI 48.6, 56.9), 53.6% had PTSD (95%CI 50.1, 57.1) and 37.1% had both depression and PTSD (95%CI 35.4, 38.8). Older women were more likely to be depressed (<0.0005). Only 22.3% of women had completed high school, but this was not associated with mental health problems. Childhood trauma was very commonly reported and was significantly associated with both depression and PTSD (at *p* < 0.001). Overall, 45.2% of women reported food insecurity, with insecurity being more common amongst those with depression and PTSD (both *p* < 0.0001).

Overall, 71.8% of the FSWs had drunk alcohol in the previous 12 months and 28.4% of FSWs indicated that they used alcohol to help them cope with being SWs. Many of those who drank alcohol consumed very large quantities, with 68.0% of drinkers having five or more drinks on a typical occasion when they drank and 52.6% drinking two or more times a week; 54.7% of drinkers reported that they considered a typical drink to be 500 mL or more. Harmful alcohol drinking was significantly more likely amongst those with depression or PTSD (both *p* < 0.0001).

Overall, 20% of FSWs indicated that they used drugs to help them cope with sex work. Women who used drugs were significantly more likely to be depressed and have PTSD (both *p* < 0.0001). Many of the FSWs experienced stigmatisation for being in sex work, and this was significantly associated with depression and PTSD. Likewise, having suicidal thoughts, which were reported to have occurred in the past month by 28.5% of FSWs, was significantly more likely for those with PTSD or depression (both *p* < 0.0001).

Overall, 64.6% of FSWs sold sex from a location outdoors. This was not significantly associated with depression or PTSD. Neither was selling in taverns or shebeens. However, PTSD was significantly more common among FSWs who sold in a brothel (PTSD *p* < 0.0001). The average number of days sex was sold was 19.1, with more days worked being associated with depression or PTSD (both < 0.0001).

In all, 81.1% of women were, or most recently had been, in relationships with a partner who was relatively or highly controlling. Being in a more controlling relationship was particularly associated with depression (*p* = 0.015). Women who had experienced physical or sexual IPV in the past year were significantly more likely to have depression and PTSD (all associations *p* < 0.001), as were women who had been raped by a man who was not a partner in the past year, i.e., a client, policeman or another man from the community.

### 3.2. Structural Equation Model

**Figure 2:**
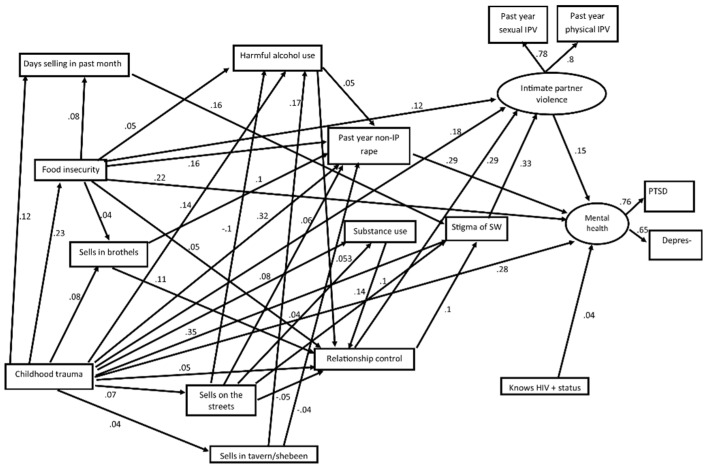
Structural equation model showing direct and mediated relationships to mental ill-health amongst female sex workers in South Africa.

The SEM showed that there were just two direct paths to the latent variable for mental ill-health, one from childhood trauma and one from knowing her HIV+ status (Figure 2 and Table 3). The model shows a myriad of indirect paths. The indirect paths were mediated by food insecurity, having a controlling partner, non-partner rape, harmful alcohol use, substance use to cope with SW, indicators of the circumstances of sex work, i.e., selling location (on streets, in taverns and brothels) and frequency of selling and experiencing SW stigma. All paths from childhood trauma had final common pathways to mental ill-health through exposure to gender-based violence (non-partner rape or intimate partner violence).

Several paths from childhood trauma to mental ill-health were mediated by past year non-partner rape. One was a direct path from childhood trauma to non-partner rape, one was also mediated by food insecurity and another by food insecurity and alcohol. They were all paths through which risk was elevated.

Other paths were proximately mediated by the three variables representing locations for selling sex (on streets, in taverns and brothels) and a further path was mediated by both selling sex in a tavern and harmful alcohol use. Overall, selling on the streets increased the risk of rape. The direct effects of selling on the street showed an elevated risk of non-partner rape, but the indirect effects, in so far as women selling on the streets drank less alcohol, were protective (*p* = 0.02). However, the total effects indicate that the protection was not enough to outweigh the overall risk of selling on the streets. Selling in taverns, however, did not increase the risk overall. One path from selling in taverns to non-partner rape had a negative sign for the direct effects, indicating that women selling in taverns were in some respects protected from non-partner rape compared to other women. The indirect effects had a positive sign, showing that the indirect pathway, mediated by harmful alcohol use, increased the risk of rape. However, this elevated risk did not remove the benefits completely, and the total effects were non-significant (*p* = 0.099) in the direction of some protection. The direct and total effects of selling in brothels were indicative of increased risk of non-partner rape.

Other paths from childhood trauma to mental ill-health were mediated by experience of intimate partner violence. One path was mediated by food insecurity. Other paths were mediated by the number of days per month selling sex and experiences of stigma, and showed increased risk of IPV; a version of this pathway was also mediated by food insecurity. Another path was mediated by selling sex on the streets and experience of stigma, increasing the risk of IPV. There was also a path from childhood trauma mediated by stigma.

Several paths between childhood trauma and partner violence were mediated by partner controlling behaviour, including one mediated by controlling behaviour and stigma experience, and another mediated by food insecurity and controlling behaviour. One path was mediated by both selling on the streets and using substances to cope with sex work, and another was mediated by only selling on the streets. The direct effects showed that FSWs selling on the streets were less subject to control by their partners than other FSWs. Another path was mediated by working in brothels, and a further one was mediated by working in taverns and harmful alcohol use.

## 4. Discussion

Our findings provide further evidence of the very high prevalence of depression and PTSD among FSWs. The factors found to influence the mental ill-health of FSWs can be grouped into six areas: their prior experiences of trauma in childhood, current food insecurity, harmful alcohol and substance use, experiences of being in a controlling relationship and gender-based violence, knowledge of HIV+ status and aspects of SW including experience of stigma, frequency of working and location of work as it impacts on risk of violence. We recognise that there are important other risk factors for depression and PTSD, particularly genetic susceptibilities and neurotrauma, but we did not have information on these [40,41].

Through the SEM, we have provided novel insights into the risk and protective factors for rape and IPV in FSWs’ working conditions, which ultimately impact on their mental health. We have shown that the number of days per month that FSWs work is related to their exposure to stigma and IPV. It seems likely that some of the IPV experienced by FSWs is an expression of the stigma of sex work, especially as sexual IPV is particularly reported by FSWs, much more so than by women in the general population. Sexual IPV was reported in the past year by 31.3% of FSWs, which is similar to their reports of past year physical IPV (37%). This pattern differs from the general population, where sexual IPV was reported by 9.1% of women and physical IPV by 2.9% [42]. It is also apparent that men who partner FSWs are more likely to be very violent, as shown in previous research [43]. We have also shown that FSWs have a variable risk of exposure to non-partner rape, depending on where they work. Women selling in taverns did not have an elevated risk of non-partner rape, notwithstanding their risk of very highly prevalent harmful alcohol consumption. However, women selling on the streets were at higher risk, even though they tended to drink less than their colleagues selling elsewhere, and FSWs selling in brothels were at a high risk of rape. These findings support the efforts made across sex work programmes in South Africa, where atypical venues, such as taverns, have seen peer educators working closely with owners to reduce the risk of violence against SWs. Furthermore, preliminary evidence from a study the authors are undertaking amongst male clients of FSWs suggest that violence against FSWs is effectively curtailed in taverns.

The variable knowledge of HIV+ status was independently impactful on mental health in the SEM, although it was not significantly associated with depression or PTSD in the bivariate analysis. All the other variables seem to have had an impact through their influence on exposure to gender-based violence—whether IPV or non-partner rape. The observation that exposure to IPV and rape have a major influence on women’s mental health, particularly the stress-related disorders depression and PTSD, is not in itself novel [22,44]. However, it is particularly noteworthy that these exposures present a common pathway through which all of the other life and work experiences had an impact, and this in itself shows the huge importance of GBV exposure in FSWs’ lives and to their mental health. This also highlights the need for structural interventions, such as decriminalisation, to enhance the security of women selling sex, particularly by reducing the sale of sex outdoors, in protecting FSW’s from GBV and, through this, protecting their mental health.

The elevated risk of poor mental health and violence stemming from exposure to childhood trauma is well described in the literature, as is that due to food insecurity, harmful alcohol use and substance abuse, and partner controlling behaviours [45]. Other trauma exposure, poverty and substance abuse are highly prevalent in our study population, and are all well recognised problems [25,43,46,47]. There are a range of strategies that may be beneficial in reducing risk. The World Health Organisation has developed the INSPIRE technical package to assist countries in developing programmes to support children exposed to trauma and to prevent violence against children [48]. Economic strengthening for families is a key intervention to reduce the risk of violence and poor mental health, as well as reducing entry to SW, as this is the more important driver in this population. South Africa has very high levels of unemployment, especially among women, and social grants are only available for women with children. Our work points to another area in which value would be reaped from a basic income grant, which has been much discussed in South Africa in recent years. Substance abuse interventions have been trialled with FSWs in South Africa for substance using women, and benefits have been found for interventions with women and their male partners, but further work is needed to investigate this for FSWs [49,50,51,52]. Other strategies that have been implemented with FSWs to reduce stigma and create a more positive narrative around sex work include community organising, for example in the Sonagachi project in Kolkata [52]. In South Africa, community organisation is seen in parts through SW organisations providing various types of support to the community. However, greater and more sustained funding is required to extend their reach and increase the programmes offered.

Sex workers in South Africa are hard to access because sex work is illegal, and they face high levels of stigma, discrimination and GBV in communities. Ideally, the sample for a national survey would be drawn from a defined population, but because we do not have this for sex work research, we believe our approach represented an innovative compromise. We acknowledge that we know little about FSWs who are not linked to sex work programmes and were not accessed through the sampling strategy. This study focused on cisgender women in sex work, because the funder had resources for research with adolescent girls and young women. We recognize that transgender women, male and non-binary SWs also face considerable mental health and other challenges, and this should be the focus of future research. This was a cross-sectional study and so the temporality of associations is in doubt. We have measures of recent prevalent mental health problems but understand that many will have been longstanding, and we have no information to help us understand when they may have first presented. The strengths of our work come from the logic underlying the associations and paths that we identified, and the fact that most were hypothesised from what is known from research and practice-based knowledge from working with FSW populations.

## 5. Conclusions

This study represents a first attempt to estimate the prevalence of depression and PTSD among FSWs linked to SW programmes in South Africa. We have confirmed that FSWs carry a substantial burden of mental ill-health. Our analysis has provided confirmatory evidence to support the idea of six influential groups of variables, stemming from the working conditions and life experiences of SWs. We have shown the contribution to mental ill-health of the location in which they sell, how much they work and the stigma they experience, as well as food insecurity, harmful alcohol consumption and substance use, knowledge of their HIV status, and their childhood experiences of trauma to their vulnerability to violence, which mediates their risk of mental health problems. Of particular interest is the finding of a particularly elevated risk of violence among FSWs selling on the streets and in brothels and a lower risk for those selling in taverns. This highlights the need for nuance in FSW programming approaches and promoting safety through attention to context.

As a result of the stigma experienced by FSWs, providing health and social care through SW programmes is essential. These need to be rooted in the SW community and adequately funded so that they can provide the needed services for FSWs’ mental ill-health and substance abuse, in addition to the more characteristic work around HIV prevention and treatment. Our work has shown that further important elements for improving mental health include protection of FSWs from exposure to violence and decriminalisation of sex work, which is foundational for enabling safer working conditions.

## Figures and Tables

**Table 1:** Key Measures.

VARIABLE NAME	ALPHA	MEASURE	DETAILS
**Sexual IPV and non-partner rape (asked separately for partner, police, clients (paying partners), other men)**		WHO violence against women [29], adapted for non-partner violence against FSWs [2]	Three items asking about being physically forced to have sex when she did not want to or agreeing to sex when she did not want to because she was threatened or afraid of what he might do, or otherwise being forced to have sex.
**Physical violence by intimate partner**		WHO violence against women [29], adapted for non-partner violence against FSWs [2]	Five items asking about: slapped, pushed, something thrown; hit with a fist or other object; kicked, dragged, beaten, choked or burnt; threaten to use or used a gun, knife or other weapon; feeling she may or would be killed.
**Food security**	0.90	Three items	Asking about having no food, sleeping hungry and going a whole day and night without eating.
**Childhood Experiences**	0.81	Childhood Trauma Questionnaire (short version) (CTQ) [30]	Five dimensions: neglect (physical and emotional) and abuse (emotional, physical and sexual); 12 items.
**Alcohol and Drug Use**	0.91	Alcohol Use Disorder Identification Test (AUDIT) C version [31]. Self-report drug use.	Measures binge drinking with volumes adjusted for the population. Drug use asked about locally relevant drugs and measure dichotomised.
**Depression**	0.90	CES-D 20 item scale [32]	CES-D scores over 20 were considered to indicate depression (i.e., a 20/21 cut point). Otherwise used as a score.
**Post-Traumatic Stress Disorder**	0.92	A short inventory (PTSD-8) [33], based upon the Harvard Trauma Questionnaire	Eight items directly linked to the DSM-IV PTSD criteria with 4-point Likert scale. A score ≥ 3 for each of the three sub-scales (intrusion, avoidance or hypervigilance) indicated PTSD. In the SEM it is used as a continuous variable.
**Relationship control scale**	0.83	Locally developed from the South African adaptation of the SRPS [34,35]	Six items designed with the community for use among FSWs. Typical item: my partner worries when I dress up that I am going to sell sex. Items summed.
**Sex work stigma**	0.74	Adapted for sex work from the People Living with HIV stigma index [36].	Five measures on a 4-point Likert scale asked about verbal, physical and sexual violence and stigma from health care workers and police. A typical question was: how often within the past year have you experienced sexual abuse because you are a sex worker. Items were summed.

**Table 2:** Characteristics of participants by mental health.

		Depression			PTSD		
Variable	Overall (n = 3005)	Yes (n = 1584)	No (n = 1421)	*p*-Value	Yes (n = 1611)	No (n = 1388)	*p*-Value
n, Age Mean (STD)	3005, 33.3 (7.95)	1584, 33.7 (7.98)	1421, 32.7 (7.88)	0.004	1611, 33.3 (7.84)	1388, 33.2 (8.06)	0.850
Education level							
Matric or higher (%)	668 (22.3)	364 (23.0)	304 (21.4)	0.336	357 (22.2)	311 (22.4)	0.933
Incomplete schooling (%)	2334 (77.8)	1219 (77.0)	1115 (78.6)		1252 (77.8)	1077 (77.6)	
n, Childhood trauma MEAN (STD) (range 0–36)	3002, 20.0 (5.79)	1584, 21.6 (5.92)	1418,18.3 (5.09)	<0.0001	1611, 21.5 (5.76)	1388, 18.4 (5.38)	<0.0001
Food insecurity							
No (%)	1643 (54.8)	681 (43.1)	962 (67.9)	<0.0001	742 (46.2)	897 (64.7)	<0.0001
Yes (%)	1355 (45.2)	901 (57.0)	454 (32.1)		864 (53.8)	490 (35.3)	
Work Circumstances							
Sells sex on the streets/outdoor location							
No (%)	1064 (35.4)	558 (35.2)	506 (35.6)	0.827	590 (36.6)	471 (33.9)	0.124
Yes (%)	1941 (64.6)	1026 (64.8)	915 (64.4)		1021 (63.4)	917 (66.1)	
Normally sells in a taverns/Shebeens							
No (%)	1914 (63.7)	975 (61.6)	939 (66.1)	0.123	994 (61.7)	914 (65.9)	0.108
Yes (%)	1091 (36.3)	609 (38.5)	482 (33.9)		617 (38.3)	474 (34.2)	
Normally sells in a brothel							
No (%)	2611 (86.9)	1350 (85.2)	1261 (88.7)	0.085	1352 (83.9)	1254 (90.4)	<0.0001
Yes (%)	394 (13.1)	234 (14.8)	160 (11.5)		259 (16.1)	134 (9.7)	
n, Days selling in past month MEAN (STD)	2988, 19.1 (7.88)	1578, 20.1 (8.00)	1410, 18.1 (7.61)	<0.0001	1606, 20.1 (7.82)	1382, 18.1 (7.82)	<0.0001
Harmful alcohol drinking and substance use							
Uses substances to enable sex work							
No (%)	2393 (79.9)	1198 (75.8)	1195 (84.5)	<0.0001	1224 (76.1)	1168 (84.2)	<0.0001
Yes (%)	603 (20.1)	383 (24.2)	220 (15.6)		384 (23.9)	219 (15.8)	
n, Harmful alcohol use score MEAN (STD)	2995, 6.32 (0.20)	1583, 6.75 (0.21)	1412, 5.84 (0.23)	<0.0001	1611, 6.89 (0.20)	1384, 5.66 (0.22)	<0.0001
Suidal thoughts and stigma							
Suicidal thoughts in the past month							
No (%)	2114 (70.5)	966 (61.0)	1148 (81.3)	<0.0001	987 (61.3)	1127 (81.2)	<0.0001
Yes (%)	883 (29.5)	618 (39.0)	265 (18.8)		622 (38.7)	261 (18.8)	
n, SW-related stigma MEAN (STD)	3000, 18.3 (5.45)	1584, 19.5 (5.69)	1416, 17.0 (4.83)	<0.0001	1611, 19.1 (5.74)	1388, 17.4 (4.95)	<0.0001
Partner controlling behaviour							
n, Relationship control MEAN (STD) (higher =more controlling)	3002, 7.8 (0.16)	1584, 8.3 (0.23)	1418, 7.5 (0.16)	0.015	1611, 8.2 (0.23)	1388, 7.6 (0.16)	0.077
Gender-based violence							
Past year non-intimate partner rape							
None (%)	1317 (44.0)	508 (32.1)	809 (57.4)	<0.0001	466 (29.0)	850 (61.5)	<0.0001
Some (%)	1676 (56.0)	1075 (67.9)	601 (42.6)		1143 (71.0)	533 (38.5)	
Intimate partner sexual violence in the past year							
None (%)	2060 (68.7)	996 (62.9)	1064 (75.1)	<0.0001	966 (60.0)	1093 (78.8)	<0.0001
Some (%)	940 (31.3)	588 (37.1)	352 (24.9)		645 (40.0)	294 (21.2)	
Intimate partner physical violence in the past year							
None (%)	1870 (62.4)	882 (55.8)	988 (69.8)	<0.0001	885 (55.1)	983 (70.9)	<0.0001
Some (%)	1127 (37.6)	700 (44.3)	427 (30.2)		722 (44.9)	404 (29.1)	
HIV							
Knows HIV+ status							
No (%)	1236 (43.0)	625 (40.8)	611 (45.5)	0.094	680 (43.4)	556 (42.5)	0.735
Yes (%)	1638 (57.0)	907 (59.2)	731 (54.5)		887 (56.6)	751 (57.5)	

**Table 3:** Female Sex Worker Mental Ill Health Vulnerability Model: Direct Effects, Indirect Effects and Total effects.

	Direct Effects				Indirect Effects				Total Effects			
Path	Standardized Coef	LCL	UCL	*p*-Value	Standardized Coef	LCL	UCL	*p*-Value	Standardized Coef	LCL	UCL	*p*-Value
Childhood trauma ->food insecurity	0.227	0.191	0.264	<0.001					0.227	0.191	0.264	<0.001
Childhood trauma ->harmful alcohol use	0.139	0.103	0.176	<0.001	0.01	−0.002	0.021	0.098	0.149	0.113	0.185	<0.001
Food insecurity ->harmful alcohol use	0.046	0.008	0.083	0.016					0.046	0.008	0.083	0.016
Sells on the street ->harmful alcohol use	−0.096	−0.131	−0.061	<0.001					−0.096	−0.131	−0.061	<0.001
Sells in taverns -> harmful alcohol use	0.167	0.132	0.202	<0.001					0.167	0.132	0.202	<0.001
Food insecurity ->past year non-partner rape	0.155	0.12	0.191	<0.001	0.006	0.002	0.01	0.006	0.161	0.126	0.197	<0.001
Harmful alcohol use ->past year non-partner rape	0.045	0.011	0.08	0.011					0.045	0.011	0.08	0.011
Sells in brothels ->past year non-partner rape	0.100	0.069	0.131	<0.001					0.100	0.069	0.131	<0.001
Sells on the street ->past year non-partner rape	0.063	0.03	0.097	<0.001	−0.004	−0.008	−0.001	0.02	0.059	0.025	0.093	0.001
Sells in taverns->past year non-partner rape	−0.035	−0.069	−0.002	0.039	0.008	0.002	0.014	0.013	−0.028	−0.061	0.005	0.099
Childhood trauma ->past year non-partner rape	0.316	0.279	0.353	<0.001	0.054	0.042	0.066	<0.001	0.37	0.334	0.406	<0.001
Food insecurity ->sells in a brothel	0.04	0.006	0.073	0.022					0.04	0.006	0.073	0.022
Childhood trauma ->sells in a brothel	0.077	0.041	0.113	<0.001	0.009	0.001	0.017	0.024	0.086	0.051	0.121	<0.001
Food insecurity ->days selling in past month	0.083	0.046	0.12	<0.001					0.083	0.046	0.12	<0.001
Childhood trauma->days selling in past month	0.121	0.08	0.162	<0.001	0.019	0.01	0.028	<0.001	0.14	0.099	0.18	<0.001
Food insecurity->relationship control	0.048	0.011	0.085	0.011	0.006	0.002	0.011	0.009	0.054	0.017	0.091	0.004
Harmful alcohol use->relationship control	0.042	0.007	0.077	0.019					0.042	0.007	0.077	0.019
Sells in a brothel->relationship control	0.108	0.071	0.144	<0.001					0.108	0.071	0.144	<0.001
Substance use->relationship control	0.142	0.105	0.179	<0.001					0.142	0.105	0.179	<0.001
Sells on the streets->relationship control	−0.048	−0.083	−0.013	0.007	0.002	−0.004	0.008	0.493	−0.046	−0.081	−0.011	0.01
Childhood trauma-> relationship control	0.052	0.01	0.094	0.015	0.034	0.02	0.048	<0.001	0.086	0.045	0.128	<0.001
Sells on the streets-> drug use	0.044	0.009	0.078	0.013					0.044	0.009	0.078	0.013
Childhood trauma-> drug use	0.075	0.038	0.113	<0.001	0.003	0.001	0.006	0.036	0.079	0.041	0.116	<0.001
Childhood trauma-> sells on the streets	0.07	0.035	0.105	<0.001					0.07	0.035	0.105	<0.001
Childhood trauma-> sells in taverns	0.036	−0.001	0.073	0.058					0.036	−0.001	0.073	0.058
Days selling in past month->stigma of SW	0.164	0.129	0.199	<0.001					0.164	0.129	0.199	<0.001
Relationship control ->stigma of SW	0.096	0.059	0.132	<0.001					0.096	0.059	0.132	<0.001
Sells on the streets->stigma of SW	0.104	0.071	0.136	<0.001	−0.004	−0.008	−0.001	0.019	0.099	0.067	0.132	<0.001
Childhood trauma->stigma of SW	0.346	0.309	0.384	<0.001	0.038	0.028	0.049	<0.001	0.385	0.347	0.422	<0.001
Food insecurity-> mental health	0.224	0.18	0.267	<0.001	0.066	0.051	0.082	<0.001	0.29	0.247	0.333	<0.001
Part year non-partner rape -> mental health	0.287	0.24	0.333	<0.001					0.287	0.24	0.333	<0.001
Violence -> mental health	0.146	0.096	0.196	<0.001					0.146	0.096	0.196	<0.001
Childhood trauma -> mental health	0.281	0.236	0.326	<0.001	0.209	0.183	0.235	<0.001	0.49	0.45	0.531	<0.001
Knows HIV+ status -> mental health	0.041	0.002	0.08	0.038					0.041	0.002	0.08	0.038
Food insecurity ->violence	0.115	0.075	0.156	<0.001	0.022	0.009	0.034	0.001	0.137	0.094	0.18	<0.001
Relationship control ->violence	0.291	0.25	0.332	<0.001	0.031	0.019	0.044	<0.001	0.322	0.28	0.365	<0.001
Stigma of SW-> violence	0.326	0.28	0.371	<0.001					0.326	0.28	0.371	<0.001
Childhood trauma ->violence	0.182	0.136	0.228	<0.001	0.177	0.15	0.203	<0.001	0.359	0.312	0.405	<0.001

note: -> indicates the direction of the path.

## Data Availability

Data are available on request from Jenny Coetzee.
